# Secoisolariciresinol diglucoside and anethole ameliorate lipid abnormalities, oxidative injury, hypercholesterolemia, heart, and liver conditions

**DOI:** 10.1002/fsn3.3250

**Published:** 2023-02-18

**Authors:** Sana Noreen, Habib‐ur Rehman, Tabussam Tufail, Huma Badar Ul Ain, Chinaza Godswill Awuchi

**Affiliations:** ^1^ University Institute of Diet and Nutritional Sciences University of Lahore Lahore Pakistan; ^2^ School of Natural and Applied Sciences Kampala International University Kampala Uganda

**Keywords:** anethole, antioxidant system, hypercholesteremia, hyperlipidemia, liver functions, secoisolariciresinol diglucoside, statin

## Abstract

Fennel seeds and flaxseed have been traditionally used against many medical ailments due to their medicinal characteristics. The aim of the study was to investigate the health properties of secoisolariciresinol diglucoside (SDG) and anethole from flaxseed and fennel seeds in rats fed with high‐fat diet. Histopathological changes in the heart and liver were also examined. Sixty rats were divided into two main groups. Group I (10 rats) was used as a negative control group and fed on the basal diet only. Group II (50 rats) was fed a hypercholesterolemic diet but not given any drugs during the trial for 2 weeks. This group was further divided into five subgroups (10 rats each). One of them was fed on the basal diet and used as a positive control group. However, the other four subgroups were fed on basal diets and anethole (20 mg/kg/day, orally), SDG (20 mg/kg/day, orally), a mixture of anethole + SDG (10 + 10 mg/kg/day, orally), and atorvastatin (10 mg/kg/day, orally) for 6 weeks. Compared to control, treatment with a combination of anethole + SDG showed a significant (*p* ≤ .05) improvement in serum levels of triglyceride (TG) (137.88 ± 1.61 mg/dL), total cholesterol‐(TC) (180.12 ± 8.99 mg/dL), LDL‐C (46.40 ± 6.67 mg/dL), VLDL‐C (11.81 ± 1.07 mg/dL), aspartate aminotransferase (AST) (75.97 ± 6.92 U/L), alanine aminotransferase (ALT) (34.83 ± 2.17 U/L), alkaline phosphatase (ALP) (130.65 ± 1.05 U/L), and malondialdehyde (MDA) (30.12 ± 1.89 mmol/g), and improved activities of catalase (70.99 ± 3.29 U/g) and superoxide dismutase (SOD) (35.13 ± 2.53 U/dL) enzymes while SDG and anethole group had relatively less impact. Atorvastatin also improved serum levels of triglyceride, total cholesterol, LDL‐C, and VLDL‐C significantly and rose serum high‐density lipoprotein cholesterol (HDL‐C) levels considerably meanwhile it had a minor but negative impact on AST, ALT, and ALP, and negligible impact on activities of MDA, CAT, and SOD enzymes compared to the positive control group. The study revealed that combining anethole and SDG may improve dyslipidemia, improve lipid profile, decrease risks of chronic heart diseases, increase HDL‐C, and enhance antioxidant enzymes' activities.

## Graphical abstract

Secoisolariciresinol diglucoside (SDG) and anethole can be explored as novel option for clinical care of hyperlipidemic patients. Secoisolariciresinol diglucoside and anethole have excellent biological properties.

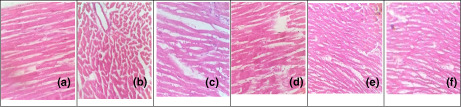

## 1 INTRODUCTION

Hyperlipidemia is a complex condition with numerous etiologies. It is characterized by elevated levels of free fatty acid, triglyceride (TG), total lipid (TL), total cholesterol (TC), and apolipoprotein B levels in the blood as well as a decrease in serum levels of high‐density lipoprotein cholesterol (HDL‐C) in hyperlipidemic patients (Rahim et al., 2023; Yao et al., 2020). It is the basic cause of atherosclerosis and heart disease risk. Worldwide, atherosclerosis greatly increases death and morbidity (Nie & Luo, 2021). High‐cholesterol diet, which results in hyperlipidemia, has a role in the onset of ischemic heart disease. In affluent nations, ischemic heart disease is the main cause of mortality (Wang & Bao, 2019). High levels of cholesterol, especially TC, TG, and LDL‐C, are mostly to blame for the emergence of cardiovascular problems, despite the fact that age, family history, hypertension, and lifestyle all play a role in heart failure (Nie & Luo, 2021). In many regions of the world, the use of medicinal plants to cure various ailments has been practiced since ancient times (Akram et al., 2021; Awuchi & Twinomuhwezi, 2021). Along with other essential medicinal herbs, fennel (*Foeniculum vulgare*) and flaxseed (*Linum usitatissimum*) have been well studied and are well known for their numerous preventive properties (Rauf et al., 2022).

The popular umbelliferous plant known as fennel can be grown as an annual, biennial, or perennial. The plant's seeds, stem, leaves, and other portions are all edible. Fennel seeds include significant levels of fat (11%), protein (10%), moisture (8%), and carbs (40%). It has minerals such as calcium, phosphorus, iron, sodium, and potassium. The vitamins, thiamine, riboflavin, and niacin, are also present in fennel. Estragole, trans‐anethole (TA), phellandrene, and fenchone are among the essential oils found in its seed; however, t‐anethole (60%–70%) makes up the majority of the extract (Rafailova et al., 2021). It is an active estrogenic medication having hepatoprotective, anti‐inflammatory, antidementia, antiplatelet, antihirsutism, antispasmodic, pain relief for primary dysmenorrhea, and anticancer effects (Palumbo et al., 2018). Fennel seed extracts are, therefore, often used in the pharmaceutical business (Saber & Eshra, 2019).

In addition to fat (41%), protein (20%), fiber (28%), phenolic compounds, a‐linolenic acid, and mucilage, flaxseed (*Linum usitatissimum*), a plant belonging to the family Linaceae, also contain other important compounds, including a range of bioactive phenols, including flavonoids, lignan, and phenolic acids (Awuchi & Okpala, 2022; Egbuna et al., 2021). SDG levels (0.6–1.8 g/100 g, or 1%–4% by weight) in flaxseed vary depending on growing method, location of plant development, and duration of cultivation (Kezimana et al., 2018). SDG are crucial for reducing atherogenic risk, hypercholesterolemia, hypertriglyceridemia, cardio‐protection, and cholesterol levels (Parikh et al., 2021). Statins are lipid‐lowering drugs that function by suppressing the production of hepatic triglycerides (Kim et al., 2022). This study aimed to investigate the potential interactions between common medications and herbal extracts used to treat hyperlipidemia. The study showed that a combination of anethole + SDG has the capacity to improve dyslipidemia and lipid profile, decrease the risk of chronic heart diseases, and increase the level of HDL‐C as well as enhance the activities of antioxidant enzymes.

## 2 MATERIALS AND METHODS

### 2.1 Experimental animals

Adult male Albino rats weighing 180–200 g, age range of 6–8 weeks, were procured from the animal house of the University of Lahore, Pakistan. Rats were kept in a clean, well‐ventilated animal home for a 12‐h light/dark cycle. They resided in six distinct groups (10 rats each) at 25–27°C. The rats were fed on a consistent schedule and provided access to flowing tap water. They were acclimatized in a laboratory for 1 week before the experiment and were provided with a basal diet and water was provided ad libitum (Aly‐Aldin et al., 2015). The procedures employed were approved by the Research Ethical Committee of the University of Lahore (IRB‐UOL‐FASH/826/2021).

### 2.2 Diet

The basal diet (g/kg diet) was composed as per the formula, i.e., 5% fat, 65% carbs, 20% protein, 5% fiber, 3.7% salt mixture, and 1% vitamin. This diet was designed to provide rats with the appropriate nutritional levels. On the other hand, the high‐fat diet (HFD) comprised 4% cholesterol (w/w) and 1% cholic acid (w/w), 24% carbs, 20.3% proteins, 5% fiber, 3.7% salt mixture, and 1% vitamin mixture (Feng et al., 2019). Basal and HFD were acquired from a Pakistani pharmaceutical company. To preserve HFD, it was kept at 4°C during its use for 2 weeks.

Moreover, flaxseed and fennel seeds were procured from the local market, Lahore. Fennel seeds and flaxseed were cleaned to remove dust particles. The herbs were then oven‐dried at 105°C for 24 h before being milled and passing through a 100‐mesh screen filter to make a powder. The powder particle size was then reduced to <0.2 mm using a grinding mill and sieves.

### 2.3 Collection of herbs extraction

The seeds were air‐dried and kept at room temperature for storage. The Soxhlet extraction assembly was used to grind the dried seeds and extract them with methanol 70% (60–80°C) for 24 h. Once the extraction process was finished, the solvent was evaporated in a rotary evaporator. The extract was then put into a clean, dry vial and kept at 4°C until further use (Natumanya et al., 2021; Yazd et al., 2019).

### 2.4 Collection of atorvastatin

Atorvastatin was obtained from a local pharmacy, Lahore, Pakistan, and ground using a mortar. The drug was administered orally once a day.

### 2.5 Kits

Activities of lipid profile (TC, LDL‐C, VLDL, and HDL) and aspartate aminotransferase (AST), alanine aminotransferase (ALT), and alkaline phosphatase (ALP) were estimated using standard kits (Merck Specialties Pvt. Ltd). Malondialdehyde (MDA), catalase (CAT), and superoxide dismutase (SOD) estimated by ELISA kit (Kamiya Biomedical Company, BT‐LAB model/Cat. No. E0560Hu) were purchased from The Gamma Trade Company for pharmaceutical and chemical.

### 2.6 Animal experiment

After 1 week of adaptation, rats (*n* = 60) were divided into two main groups. Group I (10 rats) was used as a negative control group (−ve) and fed on the basal diet only. Group II (50 rats) was fed a hypercholesterolemic diet but not given any drugs during the trial for 2 weeks (Radwan et al., 2019). This group was further divided into five subgroups (10 rats each). One of them (10 rats) was fed on the basal diet and used as a positive control group (+ve). However, the other subgroups (four subgroups) were fed on basal diets and anethole (20 mg/kg/day, orally) (Moradi Negahdari et al., 2022), SDG (20 mg/kg/day, orally) (Zanwar et al., 2014), a mixture of anethole + SDG (10 + 10 mg/kg/day, orally), and atorvastatin (10 mg/kg/day, orally) for 6 weeks (Radwan et al., 2019), respectively, as mentioned in Table 1.

**TABLE 1:** Experimental groups.

Groups		*n*	Control and hyperlipidemic rats	Treatment	Duration
Group N (−)	N_0_	10	Healthy control	Basal diet	6 weeks
Group H (+)	H_0_	10	Hyperlipidemic rats	Basal diet	2 weeks on high‐fat diet 6 weeks on Basal diet
	H_1_	10	Hyperlipidemic rats	Anethole (20 mg/kg/day, orally)	6 weeks
	H_2_	10	Hyperlipidemic rats	SDG (20 mg/kg/day, orally)	6 weeks
	H_3_	10	Hyperlipidemic rats	Anethole + SDG (10 + 10 mg/kg/day, orally)	6 weeks
	H_4_	10	Hyperlipidemic rats (standard)	Atorvastatin (10 mg/kg/day, orally)	6 weeks

### 2.7 Biochemical examination

For baseline and follow‐up testing, blood samples were taken in ethylenediaminetetraacetic acid (EDTA)‐treated tubes to evaluate hematological parameters. TC, TG, and HDL levels were measured using an enzymatic colorimetric technique described by Alkhatatbeh et al. (2019), whereas LDL‐C concentration was checked with the help of formula of Friedewald et al. (1972). The following equation was used to calculate very low‐density lipoprotein cholesterol (VLDL).

$$\text{VLDL}-c\;\left(\mathrm{mg}/\mathrm{dL}\right)=\mathrm{TG}/5$$

$$\mathrm{LDL}=\text{Total cholesterol}-\left(\mathrm{HDL}+\text{VLDL}\right)$$

### 2.8 Liver functions assay

Serum AST, ALT, and ALP were measured using an enzymatic colorimetric technique (Hafez et al., 2019).

### 2.9 Malondialdehyde assay

The content of malondialdehyde (MDA) in serum was considered as a marker of lipid peroxidation. The methods work on the basis of spectrophotometric measurements of the color generated by thiobarbituric acid (TBA) reactions with MDA. The quantity of malondialdehyde in liver tissues was evaluated as an indicator of lipid peroxidation. The weight of liver tissues was taken and then they were homogenized in ice‐cold phosphate buffer saline (PBS). One milliliter of homogenized sample supernatant was mixed with 1 mL of 10% trichloroacetic acid and 1 mL of 0.67% thiobarbituric acid. The samples were heated for 15 min in a boiling water bath before being centrifuged at 1000 *g* for 10 min. The supernatants were then collected, and the absorbance at 535 nm was measured. The concentration of MDA was then calculated and expressed as mmol/dL (Hussein et al., 2016).

### 2.10 Assessment of CAT and SOD

The technique previously reported by Hussein et al. (2016) was used to test catalase (CAT) activity in liver tissue homogenates. The technique was mentioned by Samadi‐Noshahr et al. (2021) to measure the activity of the superoxide dismutase (SOD) enzyme in liver tissues.

### 2.11 Histopathological examinations

After the rats were dissected, the liver and heart were removed and washed with a physiological solution. Samples were prepared using microscopic tissue section (Ahmed et al., 2022) and hematoxylin and eosin and they were examined by optical microscopy.

### 2.12 Statistical analysis

The results were presented as mean ± SE. A one‐way analysis of variance was used to examine the significance of differences between the control and treatment groups (ANOVA test). Differences were considered significant at a level of *p* ≤ .05 using SPSS package program (SPSS 25.00). The *p*‐value was considered significant at *p* < .05.

## 3 RESULTS AND DISCUSSION

### 3.1 Effect of anethole, SDG, and atorvastatin on the body weight of hyperlipidemic male rats

**FIGURE 1:**
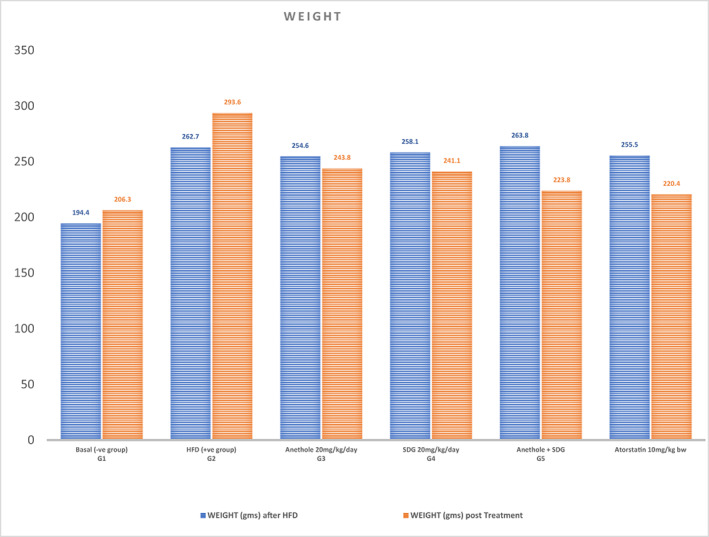
Effect of anethole, SDG, and atorvastatin on the body weight (g) of hyperlipidemic male rats. SDG, secoisolariciresinol diglucoside.

According to the data in Figure 1, the body weight of groups (2)–(6) after 2 weeks of high‐fat diet (262.70 ± 14.93 g), (254.60 ± 3.53 g), (258.1 ± 4.72 g), (263.81 ± 3.73 g), and (255.50 ± 3.97 g) significantly increased than group (1) (control group) (194.40 ± 7.80 g). The body weight of hypercholesterolemic rats considerably (*p* ≤ .05) decreased by anethole + SDG (223.80 ± 10.74 g, 40 g reduction), followed by atorvastatin (220.40 ± 33.30 g, 35.1 g reduction), SDG (241.10 ± 3.73 g, 17 g reduction), and anethole (243.80 ± 4.82 g, 10.8 g reduction) after 6 weeks of therapy compared to the positive control group (293.60 ± 8.39 g, 30.9 g increase) as compared to negative control group.

### 3.2 Effect of anethole, SDG, and atorvastatin on lipid profile among hyperlipidemic male rats

The data in Table 2 showed that rats feed with high‐fat diet (positive control group) resulted in a significant *p* ≤ .016 increase in serum TC concentration (260.03 ± 4.24 mg/dL) as well as a significant increase in TG concentrations (204.36 ± 3.12 mg/dL) as compared to negative control group (109.12 ± 3.22, 154.22 ± 1.49 mg/dL, respectively) after 2 weeks of high‐fat diet. When high‐fat diet‐fed rats were supplemented with anethole + SDG and atorvastatin, they lowered blood TC (180.12 ± 8.99 mg/dL and 163.63 ± 0.69 mg/dL, respectively) and TG (137.88 ± 1.61 and 129.37 ± 4.29 mg/dL, respectively) concentration levels considerably (*p* < .05) when compared to positive control group.

**TABLE 2:** Effect of anethole, SDG, and atorvastatin on lipid profile among hyperlipidemic male rats.

Parameter groups	Week	Basal (−ve group) N_0_	HFD (+ve group) H_0_	Anethole 20 mg/kg/day H_1_	SDG 20 mg/kg/day H_2_	Anethole + SDG 10 + 10 mg/kg/day H_3_	Atorvastatin 10 mg/kg/day H_4_
TC (40–130)	2nd	104.74 ± 2.73	260.03 ± 4.24	250.31 ± 11.19	248.90 ± 3.60	250.70 ± 7.60	251.90 ± 2.06
	6th	109.12 ± 3.22	261.41 ± 4.69^a^	214.19 ± 7.72^b^	201.40 ± 6.14^b^	180.12 ± 8.99^b^	163.63 ± 0.69^b^
TG (150–199)	2nd	153.40 ± 0.96	204.36 ± 3.12	203.60 ± 8.93	211.10 ± 4.14	212.14 ± 3.53	218.27 ± 3.48
	6th	154.22 ± 1.49	205.50 ± 3.43^a^	153.28 ± 1.76^b^	145.95 ± 9.52^b^	137.88 ± 1.61^b^	129.37 ± 4.29^b^
HDL (43–69)	2nd	43.33 ± 1.30	26.38 ± 2.71	25.94 ± 2.25	24.91 ± 1.60	25.80 ± 2.78	27.14 ± 1.61
	6th	45.03 ± 0.93	28.52 ± 3.08^a^	40.76 ± 0.99^b^	45.80 ± 1.62^b^	52.65 ± 3.35^b^	52.02 ± 5.46^b^
LDL (30–39)	2nd	29.58 ± 1.22	172.01 ± 1.28	169.76 ± 1.33	170.41 ± 3.65	167.58 ± 3.58	171.68 ± 1.62
	6th	31.66 ± 0.91	174.25 ± 2.84^a^	82.442 ± 8.95^b^	76.83 ± 4.15^b^	46.40 ± 6.67^b^	41.59 ± 4.25^b^
VLDL (11–17)	2nd	11.67 ± 0.13	34.20 ± 2.00	34.71 ± 0.41	36.42 ± 1.08	34.96 ± 1.19	34.6 ± 1.98
	6th	12.34 ± 0.51	35.08 ± 2.25^a^	21.67 ± 2.48^b^	15.66 ± 1.70^b^	11.81 ± 1.07^b^	12.66 ± 2.19^b^

*Note*: Data are mean + SEM, *n* = 10.

Abbreviations: HDL, high‐density lipoprotein; HFD, high‐fat diet; LDL, low‐density lipoprotein; SDG, secoisolariciresinol diglucoside; TC, total cholesterol; TG, triglyceride; VLDL, very low‐density lipoprotein.

^a^ Significance level: *p* ≤ .05 compared to negative control group.

^b^ Significance level: *p* ≤ .05 compared to high‐fat diet (HFD), positive control group.

Serum LDL‐C and VLDL‐C levels were substantially higher (*p* < .05) (172.01 ± 1.28 and 34.20 ± 2.00 mg/dL, respectively), while HDL‐C levels were significantly lower (26.38 ± 2.71 mg/dL) in positive control rats compared to negative control rats. High‐fat diets supplemented with anethole + SDG and atorvastatin dramatically lowered serum LDL‐C (46.40 ± 6.67 and 41.59 ± 4.25 mg/dL, respectively) and VLDL‐C levels (11.81 ± 1.07 and 12.66 ± 2.19 mg/dL, respectively) while considerably increasing serum HDL‐C levels (52.65 ± 3.35 and 52.02 ± 5.46 mg/dL, respectively) followed by anethole and SDG groups when compared to giving rats a high‐fat diet as shown in Table 1.

### 3.3 Effect of anethole, SDG, and atorvastatin on liver function of hyperlipidemic male rats

Table 3 shows serum AST, ALT, and ALP concentrations as indicators of liver function in rats. The data revealed that the positive control group exhibited significantly higher serum AST, ALT, and ALP levels (93.87 ± 1.92, 65.12 ± 1.59, and 150.45 ± 1.86 μ/L, respectively) than negative control group (46.87 ± 1.26, 23.50 ± 0.42, and 117.50 ± 1.63 μ/L, respectively). High‐fat diets supplemented with anethole + SDG significantly (*p* ≤ .05) lowered blood AST, ALT, and ALP levels (75.97 ± 6.92, 34.83 ± 2.17, and 130.65 ± 1.05 μ/L), followed by anethole and SDG group; however, atorvastatin has slightly but negligibly increased blood AST, ALT, and ALP levels as shown in Table 2.

**TABLE 3:** Effect of anethole, SDG, and atorvastatin on serum AST, ALT, and ALP among hyperlipidemic male rats.

Parameter groups	Week	Basal (−ve group) N_0_	HFD (+ve group) H_0_	Anethole 20 mg/kg/day H_1_	SDG 20 mg/kg/day H_2_	Anethole + SDG 10 + 10 mg/kg/day H_3_	Atorvastatin 10 mg/kg/day H_4_
AST (U/L) (45–80)	2nd	42.40 ± 1.07	93.87 ± 1.92	95.80 ± 1.28	95.10 ± 1.96	97.33 ± 4.32	95.18 ± 4.14
	6th	46.87 ± 1.26	96.27 ± 2.34^a^	84.50 ± 1.84^b^	82.12 ± 1.38^b^	75.97 ± 6.92^b^	97.56 ± 0.54^b^
ALT (U/L) (12–32)	2nd	21.31 ± 0.74	65.12 ± 1.59	68.52 ± 1.68	75.96 ± 1.64	73.22 ± 1.37	71.21 ± 0.69
	6th	23.50 ± 0.42	66.45 ± 1.88^a^	54.82 ± 1.53^b^	58.64 ± 0.74^b^	34.83 ± 2.17^b^	72.44 ± 0.57^b^
ALP (U/L) (57–128)	2nd	116.35 ± 0.67	150.45 ± 1.86	151.80 ± 1.30	164.76 ± 1.00	151.00 ± 0.81	151.54 ± 1.40
	6th	117.50 ± 1.63	152.28 ± 2.75^a^	138.90 ± 2.22^b^	147.26 ± 2.41^b^	130.65 ± 1.05^b^	156.08 ± 1.27^b^

*Note*: Data are mean + SEM, *n* = 10.

Abbreviations: ALP, alkaline phosphatase; ALT, alanine aminotransferase; AST, aspartate aminotransferase; HFD, high‐fat diet; SDG, secoisolariciresinol diglucoside.

^a^ Significance level: *p* ≤ .05 compared to negative control group.

^b^ Significance level: *p* ≤ .05 compared to high‐fat diet (HFD), positive control group.

### 3.4 Effect of anethole, SDG, and atorvastatin on activity of catalase enzyme, serum SOD, and serum malondialdehyde concentration among hyperlipidemic male rats

**FIGURE 2:**
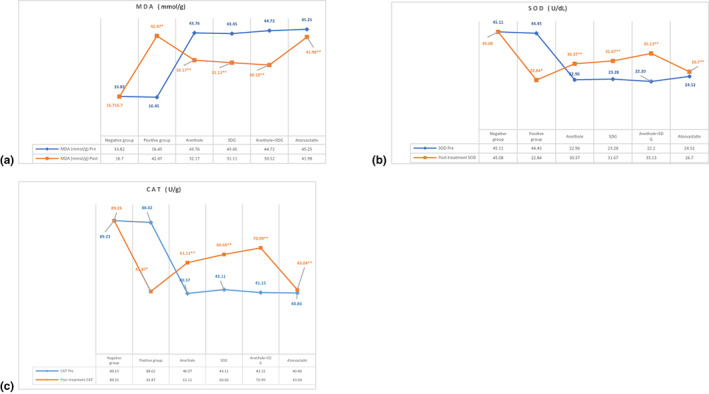
The effects of anethole, SDG, anethole + SDG, and atorvastatin on the (a) MDA, (b) SOD, and (c) CAT activities in the liver tissues of rats (*n* = 10). *Significance level: *p* ≤ .05 compared to negative control group. **Significance level: *p* ≤ .05 compared to high‐fat diet (HFD), positive control group. CAT, catalase; MDA, malondialdehyde; SOD, superoxide dismutase; SDG, secoisolariciresinol diglucoside.

Figure 2 depicts that positive control rats showed a substantial (*p* ≤ .05) rise in MDA level (42.47 ± 4.57 mmol/g) and a significant drop in CAT enzyme activity (41.87 ± 3.16 U/g) compared to negative control rats (16.70 ± 1.02 mmol/g and 89.26 ± 2.28 U/g). However, compared to positive control rats, feeding rats a high‐fat diet supplemented with anethole, SDG, and anethole + SDG considerably lowered blood MDA levels (32.17 ± 2.13, 31.11 ± 1.34, and 30.12 ± 1.89 mmol/g, respectively) and dramatically increased CAT enzyme activity (61.11 ± 2.10, 66.66 ± 3.12, and 70.99 ± 3.29 U/g, respectively) as compared to positive group, whereas atorvastatin slightly lowered MDA and inconsequentially increased CAT.

In terms of SOD enzyme activity in rat serum, tabulated findings indicated that positive control rats exhibited a substantial (*p* ≤ .05) drop in serum SOD activity (22.84 ± 0.73 U/dL) compared to negative control rats (45.08 ± 1.25 U/dL). Feeding rats supplemented diets with anethole, SDG, anethole + SDG significantly increased (*p* ≤ .05) the serum activity of SOD (30.37 ± 2.23, 31.67 ± 3.81, and 35.13 ± 2.53 U/dL, respectively) enzyme compared to the positive control group but atorvastatin had very slightly rose (*p* ≤ .05) the serum activity of SOD as shown in Figure 2.

### 3.5 Histopathological examination

**FIGURE 3:**
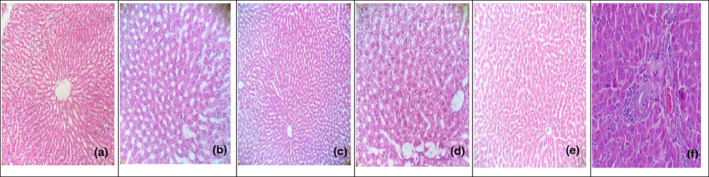
(a) Photomicrograph of liver section of negative control group shows that lobules were intact. Less than 10% of hepatocytes shows normal number of fat vacuoles in the cytoplasm. Hepatocytes are normal looking with central nuclei. Portal triads are normal (400×). (b) Liver section of positive control group shows that lobules are intact. Sixty‐three percent of hepatocytes showed fat vacuoles in the cytoplasm. Portal triads are normal (400×). (c) Photomicrograph of liver section of anethole‐treated group shows that ≈40% of hepatocytes showed moderate number of fat vacuoles in the cytoplasm. (d) Liver section of SDG‐treated group shows that lobules are intact. Less than 30% of hepatocytes show moderate number of fat vacuoles in the cytoplasm. (e) Liver section of anethole + SDG‐treated group shows that lobules are intact, and ≈25% of hepatocytes show a moderate number of fat vacuoles in the cytoplasm. (f) Liver section of atorvastatin‐treated group shows inflammatory cell infiltration in the hepatic portal space and cytoplasmic vacuolization, and ≈21% of hepatocytes degeneration was also observed.

Liver: See Figure 3.

**FIGURE 4:**
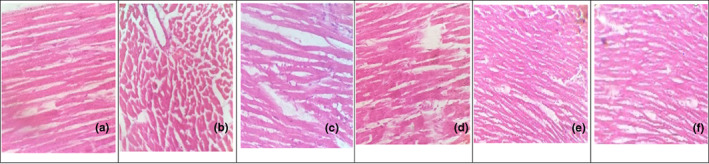
Histological pattern of heart tissue was observed. Photomicrograph of heart section of negative control rat shows normal cardiac tissue with elongated, unbranched multinucleated muscle fibers (H_X_&E 400×) as shown in figure (a). Photomicrograph of heart tissue section from rats feeding on high‐fat diet; deposition of fat was observed in the form of fatty infiltration among muscles fibers, congestion of cardiac blood vessels, and distorted striation of cardiac muscle fibers (H_X_&E 400×) as presented in figure (b). Section of heart tissue from rats feeding on anethole supplementation shows less effect as capillaries were still dilated and congested and fatty infiltration was only moderately observed (H_X_&E 400×) as compared in figure (c). Section of heart tissue from rats feeding on SDG supplementation shows minor reduction of fatty infiltration in cardiac muscles as shown in figure (d). Photomicrograph of heart section of anethole + SDG‐treated group showed a very good ameliorating effect as there was a great diminution in blood capillaries congestion, no fatty infiltration was observed and cardiac muscle fibers appeared quite normal as shown in figure (e). Photomicrograph of heart section of rat treated with atorvastatin shows less improved cardiac tissues with less distorted striation of cardiac muscle fibers (H_X_&E 400×) as presented in figure (f).

Heart: See Figure 4.

## 4 DISCUSSION

Hyperlipidemia is considered as one of the leading causes of cardiovascular disease, including atherosclerosis. It is one of the key reason for premature death internationally and is considered to become prime factor for mortality in the forthcoming time. Nutrition is widely acknowledged to play a leading role in control of hyperlipidemia and atherosclerosis (Miao et al., 2020). The term “hyperlipidemia” refers to the blood's increased or abnormally high levels of lipids and/or lipoproteins. Due to the role of cholesterol, one of the most clinically significant lipid molecules in atherosclerosis, lipid, and lipoprotein abnormalities is considered to be highly modifiable risk factors for cardiovascular disease (Islam et al., 2021; Nyarko et al., 2022). There is substantial evidence that HDL cholesterol is negatively correlated with total body cholesterol, and that lowering plasma HDL cholesterol concentrations may hasten the onset of atherosclerosis and ischemic heart disease by compromising the removal of cholesterol from the arterial wall (Kontush, 2020). According to the data, maintaining the animal on a high‐fat diet led to weight growth and dyslipidemic alterations as seen by the significantly higher blood levels of TL, TG, TC, LDL‐C, and VLDL‐C as well as a significantly lower serum HDL‐C level when compared to rats on a normal diet. These findings were supported by histological alterations in rats fed a high‐fat diet exclusively, which revealed small congested blood arteries in the heart. AL‐Aameli et al. (2019) reported that hypercholesterolemia is one of the major risk factors for coronary artery disease and atherosclerosis LDL‐C plays a crucial role in atherogenesis and it is the oxidative modification that imparts an atherogenicity to LDL. The findings of Steven et al. (2019) showed that although hyperlipidemia increases oxidative stress in the cardiovascular system, it also makes the heart and vasculature more vulnerable to stress, which were consistent with this finding. Yang et al. (2019) recently discovered that in rats fed a high‐fat diet, the development of hypercholesteremia—one of the risk factors for cardiovascular diseases—is connected with elevated blood levels of TC, LDL‐C, and VLDL‐C as well as decreased levels of HDL. Anethole's ability to decrease cholesterol may be due to the quick breakdown of LDL‐C via its hepatic receptors for eventual excretion as bile acids mentioned (Rafailova et al., 2021). The hypolipidemic action of fennel seeds and flaxseeds might be attributed due to the presence of polyphenolic compounds: tannins and flavonoids, as well as t‐anethole, the major ingredient of fennel seeds (Naderi et al., 2019; Zahnit et al., 2022). Significant antioxidant activity found in SDG in flaxseed has been associated with a decrease in TL, TC, TG, and LDL‐c levels. In hyperlipidemia, flavonoids have been shown to lower LDL and VLDL levels while raising HDL‐C levels (Alqarni et al., 2019). Many studies have reported the biological activities and therapeutic properties of phytochemicals, such as polyphenols, alkaloids, etc. (Awuchi et al., 2021, 2022; Sarvarian et al., 2022; Zahnit et al., 2022), most of which are found in flaxseed. Furthermore, compared to fennel seeds, which have lower concentrations of omega‐6 and omega‐3 but are still important in heart disease, flaxseed's hypolipidemic effect may be caused by its high content of polyunsaturated fatty acids from the omega‐3 and omega‐6, which have strong biological properties in low concentrations (DiNicolantonio & O'Keefe, 2020).

According to the current study, male albino rats receiving a diet enriched with 4% cholesterol (w/w) and 1% cholic acid (w/w) were able to develop HC, and in the second week, blood concentrations of TC, TG, and LDL‐C had significantly increased (*p* < .05). As demonstrated in Table 1, HDL‐C levels in the HCD group were lower than those in the normal group throughout both testing periods. The improved cholesterol absorption made possible by cholic acid supplementation may be responsible for these improvements. Rats fed cholesterol and cholic acid had their CYP7A1 transcripts downregulated. The rate‐determining enzyme in the liver's biosynthetic route for bile acids from cholesterol, which accounts for around 50% of daily cholesterol excretion, is called CYP7A1 (Huang et al., 2022). The increase in LDL‐C in hypercholesterolemic rats may be due to a decrease in LDL receptors or a decrease in LDL binding to its receptor in these individuals. Both the increase in blood cholesterol caused by HCD and the decrease that follows hepatic cholesterol depletion are influenced by changes in the hepatic LDL receptor (Naik et al., 2018). Current findings of histological studies also endorse this, by revealing seemingly normal histological structure of the heart in all rats treated with anethole, SDG, and their combination.

The decline in HDL‐C levels, which is linked to its crucial role in the process of reverse cholesterol transport, in which excess cell cholesterol is absorbed and processed by HDL particles before being transported to the liver for metabolism, is another risk factor for the development of atherosclerosis (Awuchi, 2021; Rehberger‐Likozar & Šebeštjen, 2015). Furthermore, reduced lipoprotein lipase activity may be the cause of elevated TG and decreased HDL‐C levels in hypercholesterolemic rats. These results are consistent with those of Radwan et al. (2019) who found that the administration of statins, such as fluvastatin, atorvastatin, and rosuvastatin, in hypercholesterolemic rats inhibits the production of cholesterol in the rat liver by blocking HMG‐CoA reductase but has no effect on intestinal cholesterol absorption. Hepatocytes become depleted of cholesterol as a result, and in response, they increase the amount of LDL‐C that is cleared from the blood by upregulating hepatic LDL‐C receptors and lowering the amount of LDL‐C that enters the circulation (Elghazaly et al., 2019).

Treatment with atorvastatin, anethole, and secoisolariciresinol diglucoside (SDG) derived from fennel seed and flaxseed all improved lipid profiles by significantly lowering TC, TG, and LDL‐C levels and significantly elevating serum HDL‐C, the latter of which became more noticeable after 6 weeks of treatment, as shown in Table 2. This observation agrees with other studies (Al‐Otaibi et al., 2020). This study demonstrated that anethole and SDG therapy may directly influence lipid metabolism, as anethole (10 mg/kg/day), SDG (10 mg/kg/day), and their combined dose (10 + 10 mg/kg/day) prevent hypercholesterolemia and hypertriglyceridemia, respectively, and reduce levels of free fatty acids and TG in hyperlipidemic subjects through their potent lipolytic activity. When compared to lovastatin, the phenolic compounds, anethole and SDG, which are present in fennel seed and flaxseed, reduce cholesterol levels in high‐fat‐fed rats via blocking hepatic HMG‐CoA reductase activity (Al‐Otaibi et al., 2020). These findings were supported by the histological study, which showed that all treated rats with anethole, SDG, their combination, and atorvastatin had what appeared to be a normal histological structure of the heart as shown in Figure 4.

A collection of clinical biochemistry laboratory blood assays called liver function tests, which also include liver enzymes, are intended to provide information about the condition of liver functions. As can be shown in Table 1, the liver transaminases (ALT and AST) and ALP in the hypercholesterolemic rats in the second week were considerably greater than those in normal animals. This may be related to hyperlipidemia leading to liver tissue damage; thus, these typically found in the cytosol of cell enzymes seep into the bloodstream when cell membranes are disrupted (Macpherson et al., 2020). High serum cholesterol level can cause liver damages (Yadav et al., 2022), and treatment for 6 weeks with anethole and SDG caused amelioration in the activity of these enzymes as shown in Table 1. The most frequent manifestation of hepatocyte injury is fatty liver, and ALT, AST, and ALP often show this. When compared to antihyperlipidemic medications, the combination of flaxseed and fennel seed may play a significant role in enhancing liver function. In rats, AST and ALT were noticeably increased, and 6 weeks of anethole, SDG, combination, and atorvastatin treatment significantly decreased their levels (AlRamadneh et al., 2022). These findings were supported by a histological examination that showed all rats treated with anethole, SDG, their combination, and atorvastatin had liver tissues that seemed to have a normal histological structure (Figure 3).

The absence of stability between the production of free radicals and the antioxidant defense actions in an organism is referred to as oxidative stress. Oxidative stress was exacerbated by hyperlipidemia when low‐density LDL‐C was oxidized. Free radicals have been implicated in the development of several degenerative conditions, such as diabetes, atherosclerosis, and cancer (Awuchi, 2021; Yao et al., 2020). According to the current study, hyperlipidemic control rats exhibited much more lipid peroxidation than normal control rats, as evidenced by an increase in MDA levels in blood. The discovery that these free radicals prevented the natural antioxidant enzyme SOD from doing its job corresponded with the increase in MDA. It is generally known that SOD and other free radical‐scavenging enzymes may shield biological systems from oxidative stress. The current study found that compared to normal rats, hyperlipidemic rats had noticeably decreased SOD and CAT enzyme activity (Chen et al., 2022). The current results of this study agreed with Samadi‐Noshahr et al. (2021), who stated that although SOD activity was reduced in hypercholesterolemic individuals, blood MDA levels increased noticeably. These results suggest that hypercholesterolemia induces oxidative stress in the myocardium, probably due to a decrease in antioxidant reserve. Reactive oxygen species levels are regulated by antioxidant enzymes like SOD and CAT (Samadi‐Noshahr et al., 2021). These antioxidants, which are created endogenously or obtained from external sources, protect cells from oxidative damage. Both enzymatic and non‐enzymatic compounds, such as phenolic and flavones, are used in the protective antioxidant processes (Hussein et al., 2016). When rats were fed a high‐fat diet supplemented with anethole, SDG, or both, blood MDA levels were much lower and CAT and SOD enzyme activity were noticeably higher than in the HFD rat group. Atorvastatin also became better. But compared to fennel seeds alone, the combination of flaxseeds and fennel seeds reduced oxidative stress and enhanced antioxidant resistance. Fennel's effect might be due to its antioxidant properties, and similar results were obtained by Gholaminejhad et al. (2017), who showed that flaxseed extract has antioxidant properties, lowering MDA levels and raising plasma SOD and CAT activity.

A previous study has revealed that stress‐induced free radical generation lowers CAT enzyme levels (Boubekeur et al., 2022; Nwozo et al., 2023). Antioxidants having free radical‐scavenging capabilities, flavonoid, and phenolic substances have the capacity to alter the physiological antioxidant status (Basharat et al., 2023; Messaoudi et al., 2022). The capacity to act as antioxidants, defend the organism against reactive oxygen species, and possibly supplant endogenous antioxidants is their most crucial characteristics (Nwozo et al., 2023). Flavonoid's biological activities are linked to their antioxidant activity via a variety of mechanisms, including “scavenging or quenching free radicals, chelating metal ions, and blocking enzyme systems that produce free radicals” (Gulcin, 2020; Nwozo et al., 2023). Flaxseed and fennel seeds show excellent antioxidant activity which helps to prevent lipid peroxidation and free radical damage; their extracts may be beneficial in the production of safe food additives (El‐shater et al., 2022).

## 5 CONCLUSION

In the current study, lipid abnormalities, oxidative injury, and hyperhomocysteinemia were induced by a high‐fat diet (HFD) while administration of anethole, SDG, and atorvastatin afforded protection against the lipidemic‐oxidative injury and was time dependent. Atorvastatin improved lipid parameters, whereas atorvastatin had slightly but negligibly increased liver enzymes and antioxidant enzymes. Anethole and SDG extract had the capability to reduce hypercholesterolemia and modulation of the oxidative stress and effects. As a result, the combination of SDG and anethole was proposed as a novel option for clinical care of hyperlipidemic patients.

## FUNDING INFORMATION

No funding was received for this study.

## CONFLICT OF INTEREST STATEMENT

The authors declare that they have no conflict of interest.

## ETHICAL APPROVAL

The study was approved by the University of Lahore, Lahore, Pakistan.

## Data Availability

Data used for this study are available on request through the corresponding author, although all the relevant data have been provided here.
